# Preparing for uncertainty during public health emergencies: What Canadian health leaders can do now to optimize future emergency response

**DOI:** 10.1177/0840470420917172

**Published:** 2020-03-31

**Authors:** Theresa W. S. Tam

**Affiliations:** 1 Chief Public Health Officer of Canada, Public Health Agency of Canada, Ottawa, Ontario, Canada.

## Abstract

It is clear that the risk for epidemics with high health and socio-economic impacts remains but there will be many unknowns at the start of future responses to these events. This article highlights principles and practices to assist health leaders in preparing for uncertainty, including integrating scalability to ensure response activities can be more easily adapted to suit evolving needs; assessing risk and capabilities to inform planning for appropriate response measures; and considering overall flexibility and adaptability of plans, systems, and resources. Ultimately, being prepared for “Disease X” is about applying the approaches that we have learned from previous events, using evidence-based practices to develop and strengthen foundational capacities, so that we are able to respond to the unanticipated in proportionate and appropriate ways.

## Introduction

Lessons from emerging health events, past and present, remind us that threats to human health are always present and will continue to be influenced by factors such as climate change, the human-animal interface, and international travel. However, emerging diseases come with many unknowns and even known diseases can behave in unexpected ways. During the initial preparation of this article, a novel coronavirus causing disease in humans (COVID-19) was emerging at the human-animal interface in China. The COVID-19 is a harsh reminder that uncertainty is part of the emergence equation and we will always be challenged to rapidly confirm the knowns and to respond as best we can, despite the unknowns. It helps to prepare with this in mind.

Severe Acute Respiratory Syndrome (SARS) was our first “Disease X” of the 21^st^ century. The World Health Organization recently coined this term to represent uncertainty as a critical planning element in preparedness for a serious international epidemic and specifically to encourage preparedness activities that account for uncertainty.^1^ There are many sources of uncertainty that are essentially the “who, what, when, where, why, and how” characteristics of a public health emergency. The “who” might be unexpected at-risk groups, such as persons with obesity who developed severe illness during the 2009 H1N1 influenza pandemic.^2^ The “what” could be unexpected outcomes such as microcephaly in infants born to mothers with Zika virus infection during pregnancy.^3^ The “where” could be an unexpected location for disease emergence, such as the H1N1 pandemic influenza that began in North America, rather than Asia, as had been anticipated and planned for.^4^ Finally, in the event of an unknown pathogen such as SARS in 2003 and now with COVID-19, the outbreak response must run parallel with a rapid gathering of international evidence (clinical, laboratory, epidemiological, etc), meaning that the level of uncertainty is dynamic and the response will need to be adjusted as and when we know more.

The purpose of this article is to identify some basic principles for dealing with uncertainty in the context of a public health emergency, to provide some examples of how these principles in combination with past experience have advanced preparedness within the health sector in Canada, and to stimulate thinking regarding what health leaders can do to further improve preparedness across the health sector in the immediate as the COVID-19 situation unfolds and going forward in the longer term.

## The role of assumptions

In terms of preparedness planning, assumptions help establish a “starting point”—a direction to quickly proceed (eg, using specific established/routine practices) until a need to adjust the course is identified. They give responders an indication of what real-time data to collect, or what to watch for, to either validate the planning assumptions or signal that a change in approach is needed. For example, an assumption used in influenza pandemic planning posits that the novel influenza virus will be transmitted from person to person in the same way seasonal influenza is transmitted. Healthcare providers will thus know what Infection Prevention and Control (IPC) precautions to utilize, which builds confidence in responders when dealing with unknowns. This starting point also helps to inform how IPC measures can be scaled up or down if reality proves to be different from the planning assumption. Therefore, assumptions are a foundational component for the development of preparedness and response plans and essential for incorporating flexibility.

## Scalability as a key principle

In the emergency preparedness and response context, scalability is used to convey the need for response activities to be dynamic. To manage demands and risks by scaling up (eg, adding more resources, enhancing active surveillance activities) or scaling down when there is evidence to indicate that specific response actions are no longer needed to achieve response objectives. A key lesson learned from past responses is that uncertainty and/or risk aversion can lead to overcompensation during a response (eg, inappropriate use of limited resources, responder burnout, or angst when trying to de-escalate). Overcompensation can be avoided by ensuring there is sufficient content in guidance, plans, and emergency exercises to demonstrate how and when the response will be scaled up or down based on risk assessments and specific data analyses that build confidence and reduce risk aversion.

## Risk and capability assessment

Another key principle involves taking a risk management approach to preparedness and response by conducting risk and capability assessments, to inform planning and response measures, and to identify gaps or enhancements that need to be addressed. Specifically, this involves making an assessment of current resources available to mitigate and respond to the risk. Assumptions can be used as a starting point to create scenarios against which risks and capabilities can be identified and assessed. For example, a planning scenario might include a person who presents to the emergency department complaining of nausea, weakness, and fever. To prepare for uncertainty, planners must consider variables in the scenario. For example, if this person had recently returned from a country with an ongoing Ebola outbreak, would the existing triage system be sufficient to identify, assess, and rapidly contain a possible Ebola case? This type of assessment helps identify specific risks, such as not including Ebola in the intake differential diagnosis for ill travellers returning from an affected area. Likewise, scenarios can facilitate a discussion regarding what capabilities are currently in place to mitigate this risk.

However, it is also important to identify what might change the risk profile in the scenario. For example, the Toronto SARS case that triggered the first hospital-based outbreak was initially missed because they had no travel history. It was only learned later that the patient had been exposed to the true index case, who did have a significant travel history but was not seen in a healthcare setting.^5,6^ This flagged a gap in our risk mitigation—that the capability to rapidly identify, and therefore contain, a SARS case that had no travel history was lacking. To close this gap, triage questions also needed to include questions about close contact with an ill traveller.

Unfortunately, not all risks can be accounted for so it is important to consider what unknowns might significantly impact a risk and what planning can be done to account for them. Emergency planners with expertise in risk and capability assessment need to work with healthcare leaders to determine how changes to risk levels will be addressed in real time, when to change course, and whether the capabilities are in place to deal with the requirements of the “new course.”

## Flexibility and adaptation

Public health preparedness efforts have been largely based on previous infectious disease outbreaks, models, and scenarios. It is important to consider the flexibility and adaptability of current plans, systems, and resources when preparing for any health emergency; this is a key principle in “all-hazard” emergency preparedness. The preparedness efforts and response resources that have been developed and used for infectious disease outbreaks are now being utilized for other public health threats.^7^ Borne out of the SARS and H1N1 experience, new federal/provincial/territorial governance structures were established to oversee the overall public health response.^8^ These governance structures have in turn been leveraged to respond to non-infectious disease national response, including most recently for the national epidemic of opioid-related deaths in Canada.

Adaptable response systems are agile enough to incorporate learnings in real time and make adjustments to response activities through feedback loops. Such systems can quickly establish new inter-sectoral connections to meet immediate specific response needs while increasing general response capabilities. Specifically, the urgency of the opioid crisis led to the mobilization of new and pooled resources that ultimately established a timely surveillance and reporting network with coroners and medical examiners. Built on an infectious disease outbreak response model, this network can potentially be leveraged for rapid mortality surveillance for emerging health events beyond opioids. There are also international efforts underway to invest in platform technologies for vaccines and therapeutics that can be adapted to target new pathogens once they are identified.^9^ Utilizing sustainable, flexible governance structures and resources ensures the response system is well exercised and able to adapt to uncertainties, while supporting readiness for other health threats and emergencies.

## Learning from past experience

The idea of identifying “lessons learned” after the conclusion of an emergency response is also a key principle of preparedness for future events. The challenge for health leaders is to ensure that the lessons identified actually translate to better understanding and ultimately an improved ability to respond. Importantly, this process has to ensure that learnings are not forgotten during peacetime or lost over time with staff turnover. The importance of risk communication, building and maintaining public trust and confidence, and cross health sector engagement are just a few of the key lessons that have been identified from past emergency responses.

The COVID-19 response is now showing us in real time the growing role of the Internet and social media in risk communication. Early, frequent communication of uncertainties is vital to building and maintaining public trust and confidence. We have learned that perception is reality and that being transparent in risk communication is essential. This means it is vital for health leaders to be forthcoming from the outset, clearly stating what we know and what we do not know, while reassuring the public that we will provide new information as and when we know it.

Despite the relatively limited spread of SARS in Canada, it served to highlight the importance of supporting and maintaining cross health sector preparedness for a seamless and comprehensive health response. This starts with increasing the number of astute frontline practitioners who are sensitized and equipped to practice “think, tell, test.” This means the first line of defence is primed to think about the possibility of an emerging pathogen, promptly tell local public health authorities, and efficiently work with clinical colleagues, hospital administrators, public health, and laboratory partners to ensure early detection and rapid containment through appropriate and timely testing.

Continuing to build and maintain a skilled and engaged workforce is essential to a robust and flexible response system. Health leaders can ensure that the lessons learned from past experiences are passed on through regular training and exercises to those entering or taking on new roles within the workforce.

Engaging across the health sector on a regular basis, in order to re-confirm roles and responsibilities, conduct joint risk and capability assessments, foster research, and provide updates on the status of preparedness activities, is also key to ensuring health sector preparedness and maintaining an ongoing state of readiness. There are operational (eg, medical evacuation and domestic transportation) and logistical (eg, supply chain and stockpiling issues) aspects of a response that require cross-sectoral engagement to address, ideally in advance of an emergency. We have seen the benefit of having contracts in place, for example, for influenza pandemic vaccine supply during the H1N1 pandemic and more recently for international medical evacuation capacity during the Ebola outbreak in West Africa.

We have also witnessed the need for advanced preparedness to engage in real-time research to ensure the response is as evidence based as possible. This has translated into advanced planning by organizations such as the Canadian Institutes of Health Research, for example, to foster rapid ethics reviews during an emergency response and supporting the Canadian Immunization Research Network to conduct vaccine clinical trials in real time.

Extending the health sector preparedness to include other sectors/disciplines (eg, social services, critical infrastructure, regulatory authorities, public safety, justice) is critical to a seamless response. Engaging communities and considering their contexts, culture, and perspectives in the preparedness for public health events is essential to the building of trust and public cooperation with health authorities during a response.

Post-SARS, emergency management practices have been adopted more widely by the health system in Canada. There is also a greater recognition of the significant social and economic impacts of public health emergencies and the importance of mitigating these impacts through mechanisms that enhance capabilities. Health leaders would be wise to ensure that emergency management, multi-sectoral coordination, and mutual aid capabilities are well integrated and exercised within their institutional response planning. Health facilities must also develop and practice their business continuity plan as a complement to their pandemic plan, given that the health of the workforce may be significantly impacted while workload demand is high.

## National capacity building for emergency preparedness and response

The Public Health Agency of Canada, established following SARS as the national coordinating body for health emergencies, has made significant investments in order to increase emergency preparedness and response capacity in Canada, build on the lessons learned from past experiences, and facilitate cross-sector preparedness and resiliency. This work has been multi-focal, ranging from the production and updating of plans, protocols, and technical guidance to conducting training, stockpiling vaccines, and therapeutics, to running exercises to test current knowledge and capabilities. Many of these efforts are intended to increase the level of preparedness across the breadth of the healthcare sector, not just public health, and not just for emergencies originating in Canada. Examples include enhancing public health laboratory and border screening capacity; establishing mutual aid and information sharing agreements^10^; and clarifying roles, responsibilities, and procedures (eg, how to request and receive aid and emergency provisions) during emergencies.^8^

Canada has met the International Health Regulations 2005 core capacity requirements^11^ and was ranked 5^th^ in the world in the Global Health Security index, assessing global health security and capabilities.^12^ Although there is a strong existing system in place, ongoing work is still needed to achieve a state of flexible and scalable readiness for the next public health emergency.

## The take-home message

As we begin a new decade, we must maintain constant vigilance as epidemics are predicted to become more frequent, more complex, and harder to prevent and contain. Health leaders need to prepare for uncertainty during an emergency response by developing, enhancing, and exercising resources—whether it be plans, people, or other resources—that can be flexible, scalable, and that are built on lessons learned and evidence-based practices.

Health leaders are well poised to see gaps and reflect on persistent challenges and recurring themes, while looking beyond their scope of influence to find creative solutions. Working from the ground up, health leaders should train staff in emergency management principles, share corporate memory, incorporate lessons learned, and build confidence through regular exercises. Exercises and training should consider the response to complex health emergencies, including pandemics, which are rapidly evolving and may last many months. Health leaders should also consider engaging with health professional regulatory bodies to explore whether and how regulatory requirements might be adapted, streamlined, or otherwise expedited during an emergency. Finally, staff must be encouraged to contribute to contingency planning by identifying concerns and repositioning them as uncertainties to be addressed.

Although it can be difficult to convince decision-makers to invest upstream in non-specific emergency preparedness resources, it is important to present the downstream benefits including risk reduction, medium- to long-term cost savings, and operational resilience. One means of fostering support is to build the understanding that investment of time, energy, and resources can pay off during normal operations, not just during large-scale health emergencies. Emergency planning can help ensure business continuity whenever there is an unexpected surge in resource demands against the backdrop of ever-limited, finite supplies. Finally, recognize that the opportunity to address critical gaps is never more urgent than during an emergency. Every event is an opportunity, given heightened political attention and investment in health capacity during a crisis. We need to build on these gains to both improve routine practice and better prepare us for future response.

Throughout this call to action to strengthen health security, I would urge health leaders to integrate a health equity lens and seek meaningful engagement from the communities they serve in order to build trust and enable a collaborative and effective response during times of uncertainty. The COVID-19 is the latest “Disease X” but it will not be the last. Health leaders, now more than ever, need to gather new knowledge, adapt response activities, and meaningfully engage with all partners across government, research, and the public at large to respond, as flexibly and effectively as possible, to this new health threat in Canada and around the world.

## References

[1] World Health Organization. Prioritizing diseases for research and development in emergency contexts. 2019 Available at: https://www.who.int/activities/prioritizing-diseases-for-research-and-development-in-emergency-contexts. Accessed November 10, 2019.

[2] Van Kerkhove MD Vandemaele KA Shinde V, et al. Risk factors for severe outcomes following 2009 influenza A (H1N1) infection: a global pooled analysis. PLoS Med. 2011;8(7):e1001053. 21750667 10.1371/journal.pmed.1001053 PMC3130021

[3] World Health Organization. Zika epidemiology update. 2019 Available at: https://www.who.int/emergencies/diseases/zika/zika-epidemiology-update-july-2019.pdf?ua=1. Accessed November 18, 2019.

[4] Public Health Agency of Canada. *Lessons Learned Review: Public Health Agency of Canada and Health Canada Response to the 2009 H1N1 Pandemic*. 2010 Available at: https://www.canada.ca/content/dam/phac-aspc/migration/phac-aspc/about_apropos/evaluation/reports-rapports/2010-2011/h1n1/pdf/h1n1-eng.pdf. Accessed November 18, 2019.

[5] Low D SARS: lessons learned from Toronto In: Knobler S Mahmoud A Lemon S Mack A Sivitz L Oberholtzer K, eds. Learning From SARS: Preparing for the Next Disease Outbreak: Workshop Summary. Washington, DC: National Academies Press; 2004:63–71. 22553895

[6] Skowronski DM Astell C Brunham RC, et al. Severe acute respiratory syndrome (SARS): a year in review. Annu Rev Med. 2005;56(3):357–381. 15660517 10.1146/annurev.med.56.091103.134135

[7] Public Health Agency of Canada. Canadian pandemic influenza preparedness: planning guidance for the health sector. 2018 Available at: https://www.canada.ca/en/public-health/services/flu-influenza/canadian-pandemic-influenza-preparedness-planning-guidance-health-sector.html. Accessed November 18, 2019. 10.14745/ccdr.v44i01a02 PMC5937063 29770091

[8] Tam T Fifteen years post-SARS: key milestones in Canada’s public health emergency response. Can Commun Dis Rep. 2018;44(5):98–101. 31007618 10.14745/ccdr.v44i05a01 PMC6449094

[9] World Health Organization. COVID 19 public health emergency of international concern (PHEIC) global research and innovation forum: towards a research roadmap. 2020 Available at: https://www.who.int/blueprint/priority-diseases/key-action/novel-coronavirus/en/. Accessed March 3, 2020.

[10] Pan-Canadian Public Health Network. Multi-lateral information sharing agreement (MLISA). 2014 Available at: http://www.phn-rsp.ca/pubs/mlisa-eng.pdf. Accessed December 17, 2019.

[11] World Health Organization. *Joint External Evaluation of IHR Core Capacities of Canada Mission Report*. 2019 Available at: https://www.who.int/ihr/publications/who-whe-cpi-2019.62/en/. Accessed December 16, 2019.

[12] Cameron E Nuzzo J Bell J *Global Health Security Index: Building Collective Action and Accountability*. 2019 Available at: https://www.ghsindex.org/wp-content/uploads/2019/10/2019-Global-Health-Security-Index.pdf. Accessed November 25, 2019.

